# Th1/Th2 Paradigm Extended: Macrophage Polarization as an Unappreciated Pathogen-Driven Escape Mechanism?

**DOI:** 10.3389/fimmu.2014.00603

**Published:** 2014-11-26

**Authors:** Eric Muraille, Oberdan Leo, Muriel Moser

**Affiliations:** ^1^Laboratory of Parasitology, Faculty of Medicine, Université Libre de Bruxelles, Brussels, Belgium; ^2^Laboratory of Immunobiology, Faculty of Sciences, Université Libre de Bruxelles, Gosselies, Belgium

**Keywords:** macrophage polarization, metabolic switch, amino acid metabolism, hypoxia, iron, PPARs, infection, immune escape strategy

## Abstract

The classical view of the Th1/Th2 paradigm posits that the pathogen nature, infectious cycle, and persistence represent key parameters controlling the choice of effector mechanisms operating during an immune response. Thus, efficient Th1 responses are triggered by replicating intracellular pathogens, while Th2 responses would control helminth infection and promote tissue repair during the resolution phase of an infectious event. However, this vision does not account for a growing body of data describing how pathogens exploit the polarization of the host immune response to their own benefit. Recently, the study of macrophages has illustrated a novel aspect of this arm race between pathogens and the immune system, and the central role of macrophages in homeostasis, repair and defense of all tissues is now fully appreciated. Like T lymphocytes, macrophages differentiate into distinct effectors including classically (M1) and alternatively (M2) activated macrophages. Interestingly, in addition to represent immune effectors, M1/M2 cells have been shown to represent potential reservoir cells to a wide range of intracellular pathogens. Subversion of macrophage cell metabolism by microbes appears as a recently uncovered immune escape strategy. Upon infection, several microbial agents have been shown to activate host metabolic pathways leading to the production of nutrients necessary to their long-term persistence in host. The purpose of this review is to summarize and discuss the strategies employed by pathogens to manipulate macrophage differentiation, and in particular their basic cell metabolism, to favor their own growth while avoiding immune control.

## Th1/Th2 Paradigm and Infectious Immunology

Historically, immunology emerged as an independent scientific discipline whose aim was to understand and possibly ameliorate the development of vaccines, a medical practice that was mostly pioneered, in an empirical fashion, by microbiologists of the end of the 19th century. Notably, however, the establishment of fundamental immunological concepts and paradigms, such as B and T cell collaboration or tolerance, was based on a reductionist (or analytical) approach that departed from a classical “host-pathogen” view of immunology, by putting much emphasis on self/non-self discrimination concepts explored with simpler protein antigens such as ovalbumin or keyhole limpet hemocyanin (KLH).

The discovery that CD4^+^ T cells can be characterized by distinct “cytokine signatures” by Mosmann and Coffman in 1986 (1) marked the great return of infection in immunology, leading to emergence of the Th1/Th2 “division of labor” paradigm in 1989, and its application to the field of vaccinology since the early 1990s. This concept led to a critical appraisal of the multiple and diverse sets of immune effectors promoting the now well established idea that the best immune response is often not the most intense nor the most specific but rather the best adapted to counteract or control the pathogen infectious cycle in the host. In consequence, identification of the nature of pathogens, selection of the appropriate immune response, repression of damaging or inadequate immune response are now recognized as crucial steps for the successful control of an infectious event. Thus, the immune reaction must be adapted to eliminate or control the pathogen and to restore homeostatic conditions during an infection to prevent severe tissue damage (2); for review see Ref. (3). The high frequency of chronic infections observed in nature demonstrates that pathogens are, however, able to manipulate these responses to evade immune control or even subvert immune reactions to their own advantage.

This review highlights current understanding of the interplay between pathogens and macrophages with a special emphasis on the role of metabolism regulation in the control of infections in mouse models unless otherwise stated. A clear understanding of the importance of macrophage polarization may reveal novel strategies for controlling infectious diseases.

## Classically and Alternatively Activated Macrophage

Myelomonocytic cells are an essential component of innate immunity and represent the first line of defense against pathogens. Myelomonocytic cells fulfill a variety of homeostatic functions, which go beyond host defense and include tissue remodeling during embryogenesis, wound healing (fibrin dissolution, removal of dead tissues, fibroblast recruitment and growth and connective-tissue remodeling), as well as orchestration and contribution to metabolic activity [reviewed in Ref. (4–6)]. Myelomonocytic cell function needs to be tailored to their tissue of residence, an adaptation that is driven by tissue-derived factors and by the physiological environment.

The myelomonocytic cell populations are particularly dynamic during inflammation or infection. Under such conditions, blood monocytes are recruited into the tissues, where they differentiate into macrophages or dendritic cells. Depending on the microenvironment, macrophages can acquire distinct functional phenotypes. The concept of macrophage polarization was first defined in 1992 with the discovery that IL-4 inhibits the respiratory burst of macrophages while enhancing expression of MHC-II and mannose receptors (CD206) on their cell surface (7). Since then, two opposite and competing phenotypes, often referred to as classically activated macrophages (M1 macrophages) and alternatively activated macrophages (M2 macrophages) [reviewed in Ref. (8–10)] have been defined and identified in several physiological settings. The M1/M2 nomenclature is derived from the Th1 and Th2 cytokines that are associated with these macrophage phenotypes.

M1 macrophages differentiate under the influence of IFN-γ and/or LPS and display functional subdivisions depending on stimulation (11). They are usually characterized by increased microbicidal activity, as witnessed by the secretion of high levels of pro-inflammatory cytokines such as TNFα and IL-6, production of reactive oxygen intermediates (ROI) and nitric oxide synthase-2 (NOS-2/iNOS)-dependent reactive nitrogen intermediates (RNI); high antigen-presenting activity and increased production of IL-12. These characteristics are promoted by IFN-γ-mediated Janus kinase–signal transducer and activator of transcription (JAK–STAT) signaling or directly by pathogen associated molecular patterns (PAMPs) such as LPS. M1 macrophages constitute the first line of defense against intracellular pathogens and promote or amplify Th1 polarization of CD4^+^ lymphocytes by IL-12 production.

M2 macrophages have been initially identified in the context of infection by helminths, differentiating from monocytic precursors under the influence of IL-4 and IL-13 produced during a strongly Th2-polarized response. M2-like cells have been described in different pathological conditions such as infections by intracellular bacteria or virus, allergy, diabetes, and cancer [reviewed in Ref. (8, 9, 12)]. They are characterized by the selective expression of markers such as arginase 1 (Arg1), chitinase-like protein (for example, Ym1), Fizz1 (Found in Inflammatory Zone 1), CD36 (fatty acid translocase), and CD206, as well as the production of low levels of IL-12 and iNOS. Depending on the context, differences in the expression of M2-associated phenotypic markers have been recognized, leading to a redefinition of this cell population to accommodate a diverse set of subtypes: M2a (induced by exposure to IL-4 or IL-13) and M2b [induced by stimulation with immune complexes, TLR, or the IL-1 receptor antagonist (IL-1ra)] macrophages drive Th2 responses, whereas M2c cells (generated by stimulation with IL-10) play a predominant role in the suppression of immune responses and tissue remodeling [reviewed in Ref. (13)].

In addition, M1 and M2 macrophages differentially express a panel of co-stimulatory receptors of the B7 family. M1 macrophages have been shown to express higher levels of CD86 and PD-L1, whereas M2 macrophages display elevated levels of PD-L2. The expression differences are less clear for CD80 (14, 15).

It should be noted that macrophages with intermediate or overlapping phenotypes have been observed *in vivo*. For example, adipose tissue macrophages from obese mice have a mixed profile, with upregulation of several M1 and M2 gene transcripts (16). Taken together, these observations suggest that the prototypical M1 and M2 phenotypes probably represent extremes of a continuum spectrum of functionally distinct cell types. In this review, we will focus mainly on the role of M1 and M2a macrophages, these latter identified on the basis of high Arg1 expression and STAT6 pathways dependence.

## Macrophage Polarization is Associated to a Shift of Metabolic Program

M1 and M2 macrophages display a drastic shift in the amino acid, glucose, lipid, and iron metabolism [reviewed in Ref. (6, 17)]. We discuss several aspects of metabolic shift in relation to pathogen growth control.

### Amino acid metabolic shift

Amino acid catabolism represents a key mechanism whereby M1 and M2-types macrophages exert their anti-microbial and immunoregulatory roles. Tryptophan and arginine degradation by innate immune cells represents the most studied examples of functional links between amino acid metabolism and immune function. This relationship is quite complex, because selected degradation of amino acids can not only affect an immune response through the depletion of important precursors to protein synthesis, but also generates new catabolites endowed with immunoregulatory functions. In keeping with the general purpose of this review, we will mostly discuss herein the known links between expression and function of amino acid catabolizing enzymes and M1/M2 differentiation.

Among the three different enzymes, indoleamine 2,3-dioxygenase 1 (IDO1), 2 (IDO2), and tryptophan 2,3-dioxygenase (TDO), which catalyze oxidative Trp catabolism, IDO1 represents the best known and widely studied example illustrating how amino acid metabolism and immune regulation interface. IDO1 has been shown to confer potent anti-microbial activities *in vitro*. Through the depletion of the essential amino acid tryptophan, IDO1 can restrict *in vitro* the growth of a wide range of pathogens, including viruses, bacteria, and protozoa (18–22). Although IDO1 is constitutively expressed by numerous tissues including the epididymis, uterus, spleen, small intestines, and prostate (23), its expression is highly inducible by both type I and type II interferons, with IFN-γ considered as one of the strongest inducers of IDO expression and activity. As a consequence, and despite the fact that IDO1 expression is not restricted to hematopoietic cells, some authors have come to consider IDO1 as a prototypical M1 marker (11).

The role of IDO1 as potent anti-microbial agent *in vivo* remains, however, questionable, due in particular to the well-known immunosuppressive function of IDO1-expressing cells. Increased tryptophan catabolism has been indeed observed in the tumor microenvironment and in the placenta, contributing to tolerance to, respectively, tumor and fetal antigens. Although apparently at odds with the anti-microbial properties of IDO1, recent observations seem to concur with the notion that despite its association to an M1-like state, IDO1 may indeed primarily mediate anti-inflammatory/regulatory roles *in vivo* opposing pathogen clearance. Indeed, genetic IDO1 ablation or pharmacological inhibition of IDO reduced parasitic load in *Leishmania major* infected mice (24). Similarly, *in vivo* administration of a pharmacological inhibitor of IDO to *Toxoplasma gondii* infected mice or infection of an IDO1-deficient mouse strain led to a more efficient control of parasite growth by IDO1-impaired hosts (25). In keeping with this prevalent immunoregulatory role of IDO1 *in vivo*, inhibition of IDO1 activity enhanced both CD4 and CD8 T cell responses to influenza virus infection, globally improving the antigen-specific memory response to this virus (26, 27).

Recent observations, albeit performed exclusively *in vitro*, have shed a new light on the possible role of IDO1 in the M1 vs M2 differentiation pathways (28). In this study, the authors demonstrate that forced expression of IDO1 in the human acute monocyte leukemia cell line THP-1 is sufficient to induce an M2-like profile, characterized by high IL-10 and low IL-12 expression pattern. Similarly, siRNA-mediated inhibition of IDO1 expression led to an increased expression of M1 markers, suggesting an important and cell autonomous role for IDO1 in regulating macrophage polarization. Collectively, and although the molecular and cellular mechanisms need to be identified, the available evidence suggests the existence of negative feedback regulatory loop whereby expression of IDO1 under M1-polarizing conditions contributes to the attenuation of a T cell mediated inflammatory response while favoring a M1 to M2 shift in macrophages.

Elevated arginine catabolism has also been linked to immunoregulation and anti-microbial immunity. Noteworthy, two of the prototypic M1 and M2 markers (respectively, iNOS and Arg1) have the capacity to use l-arginine as a substrate, leading to the production of l-citrulline and nitric oxide (NO), or l-ornithine and urea, respectively. The finding that concentrations of l-arginine at site of inflammation often decline to undetectable levels suggests an important role for l-arginine catabolism during an immune response (29, 30).

**Figure 1:**
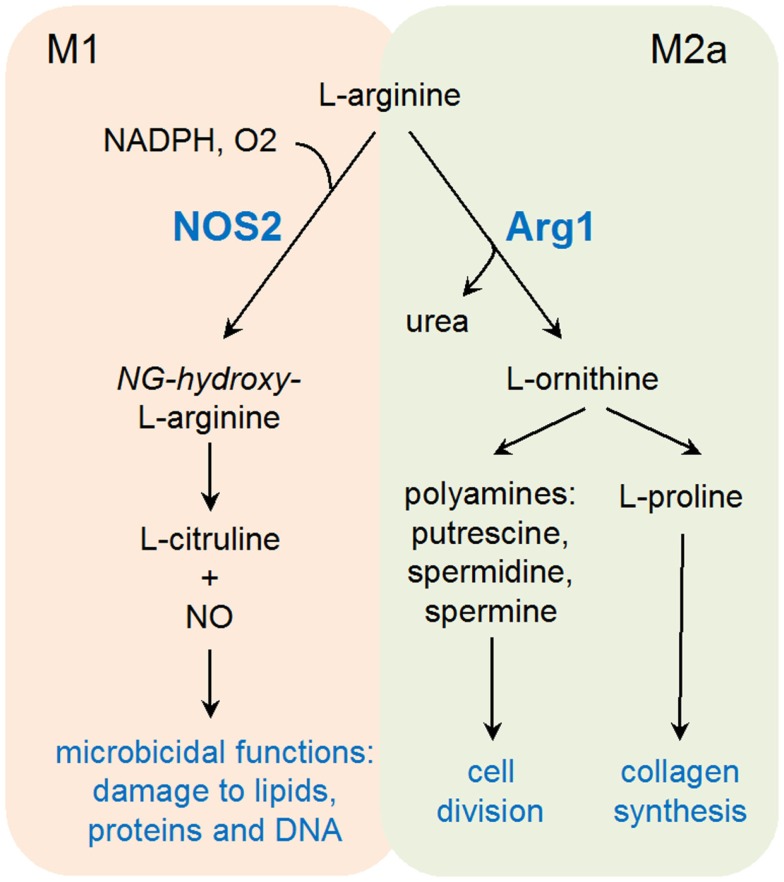
**Schematic representation of l-arginine catabolism in M1 and M2a-type macrophages**.

Products of Arg1 vs iNOS appear to fulfill diametrically opposed functions (Figure 1). Arg1 enhances collagen synthesis and cell growth via l-ornithine production, while iNOS opposes cell viability and proliferation. Moreover, iNOS protein translation appears as particularly sensitive to l-arginine levels, providing a further mechanism, in addition to competition for the same substrate, of counter-regulation between these two enzymes (31). This substrate competition between iNOS and Arg1 has been extensively described in mouse models (32), and further studies may be required to validate the universality of this concept in other species. Arginase activity has been found in lesions of patients with cutaneous leishmaniasis and in human tuberculous granulomas, suggesting an evolutionary conserved response linking arginine metabolism to infection (33–35).

M1-derived NO represents a major effector molecule in macrophage-mediated cytotoxicity, playing an important role in controlling bacterial and parasitic infections (36–38). In keeping with the previously discussed antagonistic role of Arg1 and iNOS, induction of arginase activity represents an efficient immune escape strategy developed by several pathogens. As an example, active induction of Arg1 gene transcription in infected macrophages through manipulation of the STAT6 signaling pathway has been demonstrated for both *T. gondii* and *Leishmania donovanii* (39–41). The discovery that T cells are particularly sensitive to local arginine concentrations has further confirmed the important immunoregulatory role of Arg1, able therefore to inhibit both innate (NO production) and adaptive immune responses to parasites (30, 32). In addition to its immunoregulatory role, increased arginine metabolism has been shown to directly promote intracellular growth of several *Leishmania* species, notably through the accumulation of polyamines (42, 43). A compelling argument in favor of a role of arginine catabolism in promoting parasite growth is the expression of endogenous arginase by several parasites including *Leishmania, Crithidia*, and *Leptomonas* [see Ref. (44) for review].

Most of the available evidence links therefore Arg1 expression, and thus the M2 phenotype, to a disease-promoting activity. Noteworthy, however, expression of Arg1 in macrophages also plays an important role in protecting the host against the lethal effects of chronic Th2 pathology, as recently demonstrated in mice infected by *Schistosoma mansoni* (45). A similar role for Arg1 in suppressing pathologic inflammation has been derived from studies conducted in acutely *S. mansoni*-infected Arg1-deficient bone marrow chimeric mice (46). Finally, the importance of Arg1 in wound healing has been recently demonstrated using both pharmacological inhibition of arginase activity and genetic deletion of Arg1 in both haematopoietic and endothelial cells, further illustrating the important and positive role of this enzyme in tissue repair and homeostasis (47).

Although expression of Arg1 is often associated to an immunoregulatory/pathogen-growth promoting setting, recent studies have uncovered a previously unsuspected effector role for this enzyme against nematode infection. In models of secondary infections, arginase activity was found to play a key role in the acquired protective immunity against reinfection with an intestinal helminth. Upon primary exposure to *Nippostrongylus brasiliensis*, mice developed the capacity to retain larvae in the infected skin, thus reducing the worm burden in the lungs (48). In this model, Arg1 was shown to play a key role in larvae trapping, confirming previously suspected antiparasitic effects of arginase activity against *Heligmosomoides polygyrus* (49). The capacity of the arginine catabolite l-ornithine to inhibit *H. polygyrus* larvae mobility suggests a plausible mechanism whereby Arg1 may protect the host during parasitic infections (50).

In summary, catabolism of both tryptophan and l-arginine lays at the heart of the M1/M2 dichotomy. Arginine catabolism represents an important immune mechanism limiting pathogen growth and spread, exploited by both M1 (through iNOS) and M2 (via Arg1) – like macrophages. Through distinct and mutually exclusive enzymatic reactions, the same amino acid can therefore be exploited to generate effector molecules tailored to different pathogens. Moreover, both iNOS and Arg1 have also been shown to dampen excessive inflammatory and T-cell mediated reactions. Whether these counter-regulatory properties have been evolutionary selected to control unwanted immune activity or represent unintended side effects remains to be established. In marked contrast, it is difficult at present to evaluate the precise role of IDO1 as an anti-microbial effector. Despite a large body of *in vitro* data, a role for IDO1 in limiting pathogen growth *in vivo* is still lacking, suggesting that despite being associated to an M1-like subpopulation of cells, tryptophan catabolism may primarily serve a regulatory role *in vivo*. In conclusion, amino acid catabolism elegantly illustrates the dual function of M1 and M2 macrophages, both subsets being able to express anti-microbial and immunoregulatory functions.

### Hypoxia

Decrease in oxygen pressure (hypoxia) in tissues can result from various causes such as mechanical or infection induced inflammation, intense metabolic activity but also obesity and tumor growth [reviewed in Ref. (51)]. The transcription factor hypoxia-inducible factor-1 (HIF-1) is a central mediator of hypoxic adaptation. In normoxia, HIF-1 is repressed primarily through the action of a family of hydroxylases, which targets HIF-1 subunits for degradation in an oxygen-dependent manner. In hypoxia, HIF-1 is rapidly stabilized in cells and induces the expression of hundreds of genes, which regulate angiogenesis, metabolism, growth and survival (50).

In 1938, Kempner was the first to associate cellular metabolism and inflammation: he examined the chemical composition of control or inflamed tissues and reported chemical changes that he interpreted as mainly due to the aerobic glycolysis of blood cells (52). There is indeed growing evidence that tissue foci of inflammation display a declining oxygen gradient, as compared to oxygen-rich blood stream, leading to increased cellular HIF-1α levels particularly in phagocytes, and activation of genes required to maintain viability and activity in these demanding conditions (low oxygen and glucose). It is noteworthy that HIF-1α may also exert regulatory effects in normoxia, in particular during bacterial infection, where HIF-1α expression is stimulated through TLR receptor engagement and cell signaling pathways (such as NF-κB and MAPK) [for review see Ref. (53)].

**Figure 2:**
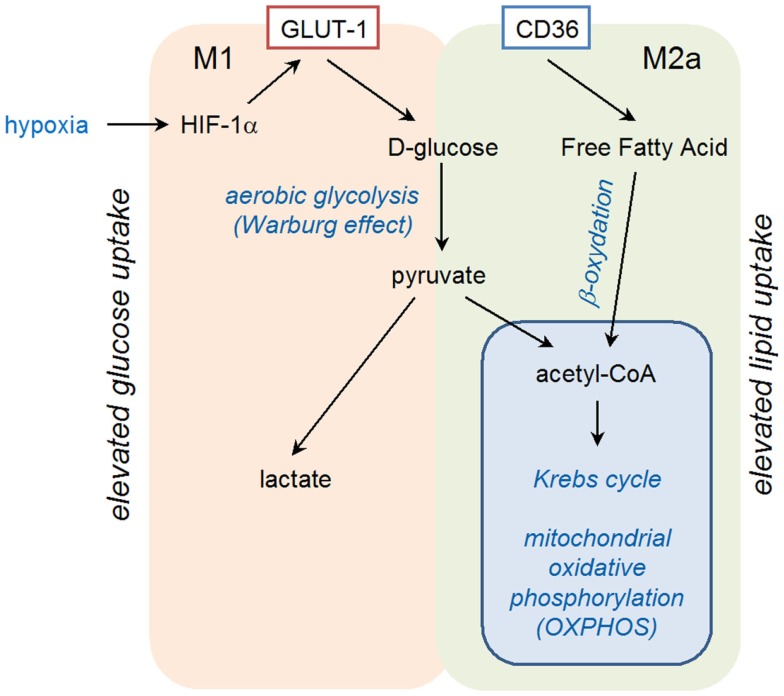
**Schematic representation of polarized metabolism in M1 and M2a-type macrophages**.

Exposure of primary human monocyte-derived macrophages to hypoxia for 16 h has been shown to result in increased mRNA levels for vascular endothelial growth factor (VEGF), glucose transporter (GLUT-1), and matrix metalloproteinase 7 (MMP-7) (54). Induction of VEGF in hypoxic conditions is involved in the pro-angiogenic activities of macrophages in tumors, whereas GLUT-1, a glucose transporter, may favor macrophage survival in ischemic tissues by increasing glucose uptake for glycolytic production of energy (Figure 2). The increase in mRNA coding for MMP-7 could have several consequences, as MMP-7, the smallest MMP, is able to digest many components of the basement membrane and extracellular matrix, and is involved in proteolytic processing, leading to activation of TNF-α, defensins, and other MMPs.

A number of reports have shown that hypoxia profoundly affects macrophage recruitment and development of M1 associated anti-microbial functions. Specific deletion of HIF-1α in the myeloid lineage resulted in lower ATP levels caused by decreased glycolysis. This metabolic defect results in profound impairment of myeloid cell aggregation, motility, invasiveness, and bacterial killing (55). Analysis of intracellular killing of Group B streptococci by bone marrow-derived macrophages revealed approximately sevenfold more viable bacteria within the HIF-1α deficient macrophages as compared with the wild-type cells. *In vivo*, loss of HIF-1α in myeloid cells impaired infiltration at the dermal-epidermal border of inflamed skin and prevented resolution of passively induced arthritis. HIF-1α agonist mimosine and the pharmacological agent AKB-4924 (which stabilizes HIF-1) were both shown to enhance the antibacterial activity of phagocytes and to kill the pathogen *Staphylococcus aureus in vitro* and *in vivo* (56, 57). Conversely, mice lacking HIF-1α in their myeloid lineage developed larger necrotic skin lesions upon subcutaneous infection with group A *Streptococcus* (58). Murine macrophages exposed to low oxygen tensions have been shown to display M1 phenotype as suggested by increased antigen-presenting and phagocytic functions that involved IFN-γ production (59).

Collectively, these observations indicate that HIF-1α critically regulates pathways essential for the maintenance of energy homeostasis and function of macrophages in sites deprived of oxygen. However, a recent report contradicts this conclusion, as the authors found that hypoxia in *L. major* skin lesions impaired the NO-dependent leishmanicidal activity of macrophages in a reversible manner. The mechanism was found to involve impaired translation of NOS protein (60). This latter study is in accordance with two recent reports showing (i) that tumor-infiltrating macrophages in hypoxic areas displayed an altered pro-tumoral phenotype, characterized by impaired M1-type function (61) and (ii) that tumor-derived lactic acid induced *vegf* and *Arg1* and the M2-like polarization of tumor-associated macrophages in a HIF-1α-dependent manner (62).

### Iron metabolism

Iron is an ideal redox catalyst, accepting or donating electrons, implicated in numerous cellular processes such as respiration, DNA replication but also host defenses by the production of reactive oxygen and nitrogen intermediates. Almost all living organisms from archaea to eukaryotes display absolute requirement for iron [reviewed in Ref. (63–65)]. Erythropoiesis is the most avid consumer of iron in the mammal organism as erythrocytes contain approximately 1 billion of ferric atoms. Approximately 60–70% of the human adult body iron is bound within hemoglobin (2.5 g). Macrophages play a central role in regulating iron metabolism since they recycle heme iron from senescent erythrocytes and regulate its storage. On the other hand, microbes have evoked multiple strategies to utilize iron because a sufficient supply of this metal is linked to pathogen proliferation, virulence and persistence. The expression of iron uptake systems is linked to virulence in a broad range of pathogens including bacteria, protozoa and fungi. The control over iron homeostasis is thus of central importance in host–pathogen interaction, in which both opponents compete for iron. A complex network of host proteins renders this valuable nutrient largely inaccessible to pathogens, a concept usually known as “nutritional immunity.” Of course, control of intra- and extracellular pathogens requires distinct mechanisms of iron restriction in different compartments. M1 macrophages, by repressing ferroportin (a cellular iron exporter) and CD163 (a hemoglobin scavenger receptor) and inducing ferritin (which favors iron intracellular sequestration and storage), reduce the labile iron pool (LIP), the metabolically active fraction of cytosolic iron that is available for metabolic purposes. In contrast, through the upregulation of ferroportin and the downregulation of ferritin, M2 macrophages have reduced iron storage and enhanced release of iron (66). Sequestration of iron by M1 macrophages would have a bacteriostatic effect (since iron is essential to support bacterial growth) and thus represents a host protective response. Conversely, iron release from M2 macrophages would favor tissue repair. Interestingly, iron is also an important regulator of immune effector functions, immune cell proliferation and cytokine production. Iron antagonizes the IFN-γ-induced expression of MHC-II, iNOS, and TNF-α and shifts the Th1/Th2 differentiation toward a Th2 reaction.

### Lipid oxidative metabolism

Peroxisome proliferator activated receptors (PPARs) are lipid-activated transcription factors of the nuclear hormone receptor superfamily. Endogenous ligands that regulate the PPAR family remain poorly characterized and include free native and modified fatty acids. PPARs regulate expression of target genes as obligate heterodimers with the retinoic X receptors (RXRs). In mammals, there are three PPAR isoforms (PPARα, -γ, -δ) expressed in a large variety of cells and playing pleiotropic functions in fatty acid metabolism including transport, synthesis storage, mobilization, and oxidation [reviewed in Ref. (67)].

**Figure 3:**
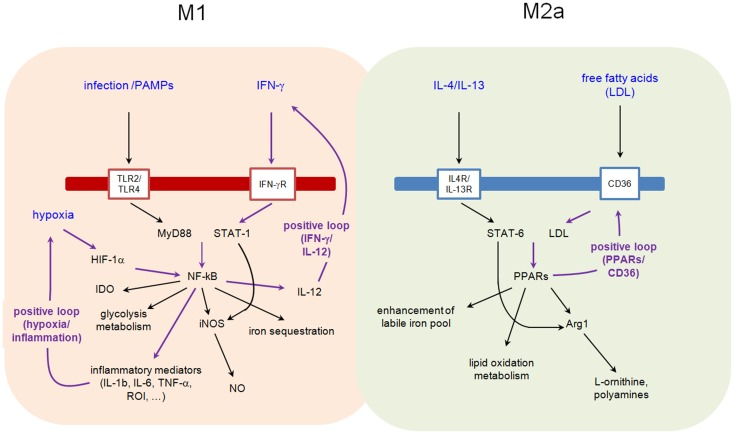
**Interplay between metabolic pathways and M1 and M2a functions**.

Binding of IL-4 or IL-13 to their cognate receptors, IL-4Rα/IL-2Rγ and IL-4Rα/IL-13Rα1, respectively, initiates a cytoplasmic signaling cascade leading to tyrosine phosphorylation of transcription factor STAT6. Phosphorylated STAT6 dimerizes and translocates to the nucleus, where it induces the expression of numerous target genes. Among them, PPARγ, PPARδ, and PPARγ-coactivator-1β (PGC-1β) appear particularly important. PPARγ mediates the expression of the main M2 phenotype markers such as Arg1 and CD36 (favoring the uptake of fatty acid and apoptotic cells) and increases oxidation metabolism of fatty acid [reviewed in Ref. (68)] (Figure 2). By its transrepressive action, PPARγ blocks the expression of numerous pro-inflammatory mediators induced by LPS and IFN-γ such as IL-1β and iNOS. Although PPARγ is not essential for monocyte/macrophage differentiation, it functions as an important modulator of macrophage lipid metabolism and a fine-tuner of immune functions (Figure 3). Whereas the instructions for alternative macrophage activation are provided by IL-4 and IL-13, the acquisition and long-term maintenance of this phenotype implicate PPARγ and PPARδ. PPARγ KO mice are deficient in M2 macrophages, develop spontaneous chronic Th1 inflammation in lung (69) and are more susceptible to obesity and insulin resistance, suggesting that homeostatic functions performed by M2 macrophages might allow animals to more efficiently store and oxidize incoming lipids, thereby maintaining insulin sensitivity and glucose tolerance by attenuating inflammation (70).

Reciprocally, fatty acids facilitate the acquisition and maintenance of M2 phenotype (Figure 3). In a PPARδ-dependent manner, monounsaturated fatty acids, such as oleic acid, were found to synergize with IL-4 to enhance the expression of alternative activation signature genes such as Arg1 in macrophages. Arg1 promoter is directly activated by PPAR/RXR heterodimers [reviewed in Ref. (67)] providing a molecular explanation for the observed synergy between nutrients and Il-4/IL-13 signaling pathways.

### The energy sensors: AMPK and mTOR

The AMP-activated kinase (AMPK) represents a well described energy sensor able to adapt cell metabolism to available substrates and/or cellular energetic requirements. Accumulation of cellular AMP, indicative of a reduction in the energy status of the cell, activates the AMPK, which in turn will phosphorylate a series of substrates to favor metabolic reactions (such as stimulation of mitochondrial biogenesis, oxidative metabolism and autophagy), while decreasing ATP consuming, biosynthetic pathways. The mammalian target of rapamycin (mTOR) plays a similar role, by adapting metabolic reactions (such as protein synthesis) to nutrient availability in order to preserve cellular homeostasis. Pathogens have been shown to manipulate these regulatory pathways to highjack cell metabolism to their own benefit [reviewed in Ref. (71, 72)]. Collectively, these studies have highlighted how, by inhibiting AMPK and/or maintaining an activated mTOR pathway, pathogens generate essential substrates for their own replication. Notably, a link between macrophage metabolism and inflammation has also been recently established. A reduction in macrophage AMPK activity is associated with M1-like, pro-inflammatory status, while increased AMPK activity promotes an anti-inflammatory, M2-type, response [for review see Ref. (73)]. Inhibition of AMPK expression or activity promotes expression of pro-inflammatory mediators in macrophages activated by LPS (74). Similarly, AMPKα1 KO macrophages failed to acquire an M2 phenotype, retaining high levels of iNOS expression even when stimulated in the presence of M2-polarizing factors such as IL-4 (75).

In summary, polarization states and functional properties of macrophages largely depend on environmental conditions, such as hypoxia, cytokines, pathogen-derived TLR-ligands, and lipid mediators. Metabolism shift associated to macrophage polarization appears highly adapted to microbicidal and tissue regeneration function of M1 and M2 macrophages, respectively.

## M2-Like Polarization as a Pathogen-Induced Immune Escape Strategy

The interaction between pathogenic microbes and their host is determined by survival strategies to compete for essential nutrients. During evolution, microbes have developed strategies to access selected nutrients from the host, while these, in return, have devised defensive means to restrict their availability to pathogens. Long-term pathogen persistence is frequently the consequence of a subtle equilibrium between the nutritive needs of host and pathogens. M2 macrophages appear as a favorable niche for long-term persistence of numerous intracellular pathogens. Some evidences suggest that intracellular pathogens can induce or benefit from PPAR expression and/or high iron availability in infected macrophages.

*Mycobacterium tuberculosis*, the agent of tuberculosis, is well adapted to survive within the hostile macrophage environment. Current evidence (76) indicates that *M. tuberculosis* and BCG infection causes a time-dependent upregulation of PPARγ expression in infected macrophages. Even uptake of dead bacteria triggered PPARγ expression in macrophages, suggesting a pattern recognition receptor-mediated triggering mechanism. Accordingly, recent studies have shown the implication of TLR2 (77) and mannose receptor (CD206) (78) in this process. PPARγ expression and activation led to increased lipid droplet formation, expression of M2 markers and downmodulation of bactericidal M1 response including respiratory burst and NO production. Involvement of CD36 in lipid droplet formation was further confirmed by decreased BCG-induced lipid droplet formation in CD36 deficient macrophages (77). These observations suggest that PPARγ expression may not only represent an escape mechanism to circumvent the protective host response, but may also provide the nutrient rich environment required for mycobacterial growth and survival by promoting host lipid metabolism.

A key anti-pathogen effector of M1 macrophages is NO, which is involved in direct killing of pathogens, such as *M. tuberculosis*. Interestingly, a report has shown that Arg1 was induced in macrophages *in vitro* via a STAT6 independent and TLR dependent pathway, and prevented NO production. The absence of Arg1 was associated with increased NO expression and enhanced control of mycobacteria. These observations suggest that chronic infection could be linked to pathogen-induced Arg1 expression (37). Conversely, abrogation of Arg1 expression in hypoxic macrophages where iNOS was rendered ineffective resulted in exacerbated lung granuloma pathology and bacterial burden, suggesting that Arg1 could also play a positive role in the control of *M. tuberculosis* (79).

Iron availability in macrophage is also crucial for *Mycobacteria* growth (64, 65). Iron overload enhanced susceptibility while iron-poor diets reduced *Mycobacteria* virulence in mice (80). The depletion of intracellular iron occurs in M1 polarized macrophages. However, *Mycobacterium* produces siderophores (mycobactins) with incredible high affinity to iron able to remove iron from transferrin and lactoferrin, leading to augmentation of iron concentrations in infected macrophages favoring *Mycobacterium* growth. As previously mentioned, intracellular iron accumulation could also downregulate M1 phenotype by negatively affecting iNOS expression (81).

*Brucella* spp. are facultative intracellular α-proteobacteria and the causative agent of the world’s leading zoonotic disease brucellosis (82). Its pathogenesis is mainly based on its ability to survive and multiply inside macrophages. Treating *Brucella abortus*-infected mice with a PPARγ inhibitor led to a significant decrease in splenic colonization during the chronic phase of infection (83). In contrast, treatment of *B. abortus*-infected mice with PPARγ agonist led to increased expression of M2 markers in splenic macrophages and enhanced bacterial count in the spleen during the chronic infection phase (83). These results strongly suggest that pathways downstream of PPARγ contribute to generate a niche for persistence of *B. abortus*. In the same study (83), further analysis of *in vitro* infected macrophages suggested that PPARγ favors intracellular glucose availability for *Brucella* by shifting the energetic cell metabolism from a typical M1-associated glycolytic pathway to a glucose-sparing lipid oxidation associated to M2 differentiation.

Although iron is needed by bacteria for growth, it also plays an important role in the anti-microbial M1 activities of macrophages such as generation of hydroxyl radicals. *Brucella* appears well equipped to deal with the iron-poor environment in M1 macrophages. *Brucella* expresses two siderophores [2,3-dihydroxybenzoic acid (2,3-DHBA) and 2,3-DHBA-based molecule brucebactin] and a heme oxygenase (BhuO) allowing the use of heme as an iron source [reviewed in Ref. (84)]. It has been observed that iron supplementation increased the ability of macrophages to control intracellular *Brucella* (85) suggesting that iron chelation may represent a valuable strategy whereby this bacterium impedes the generation of toxic hydroxyl radicals.

*Salmonella* are Gram-negative bacteria infecting hosts via the gastrointestinal tract. Host-adapted strains of *Salmonella enterica* cause systemic infections (typhoid fever) and have the ability to asymptomatically persist within host tissues for long periods of time in 1–6% of patients [reviewed in Ref. (86)]. Early protective Th1 responses are followed by pathogen-permissive Th2 responses. *Salmonella* infection caused accumulation and persistence of hemophagocyte macrophages (characterized by the ingestion of non-apoptotic cells) expressing M2 markers (CD36 and CD206) in spleen (87). During later stages of infection *in vivo, Salmonella* was found preferentially associated with these cells (88), possibly representing a specific niche for persistent infection. PPARδ was found upregulated in *Salmonella* infected macrophages *in vitro*. PPARδ deficiency dramatically inhibited *Salmonella* replication *in vitro* and *in vivo* and its pharmacological activation enhanced bacterial growth in mice. Absence of PPARδ was associated to a decrease of available glucose necessary to *Salmonella* persistence in macrophages (88). How macrophages acquire and/or maintain an M2 phenotype *in vivo* during *Salmonella* infection is presently unknown.

*Leishmania* represent obligate intracellular protozoan parasites that are transmitted in the dermis of the mammalian host by blood-feeding sand flies. After transmission, these parasites invade mainly macrophages, which are decisive effector cells that either kill or host the intracellular parasites depending on the balance of iNOS and Arg1 [reviewed in Ref. (89)]. Control of parasite growth was associated with the induction of strong Th1 responses and induction of iNOS-expressing M1 macrophage. Accordingly, there was a clear direct correlation between the parasite load and the arginase activity in the lesions. Inhibition of arginase activity during the course of infection reduced parasite growth (43). *In vitro*, parasitized dendritic cells showed coordinated transcriptional modulations that correlated in part to PPARχ upregulation and promoted the generation and storage of neutral lipids, such as triacyl-sn-glycerol and cholesteryl esters (90), that can be important for the synthesis of key parasite membrane components. Accumulation of neutral lipid has been also shown to reduce antigen processing and presentation to effector T cells (91). *Leishmania* parasites are coated with phosphatidylserine (92), a major surface characteristic of apoptotic cells (93) recognized by CD36 expressed by M2 macrophages (94). As engulfment of apoptotic cells by CD36 pathways leads to induction of PPAR, it is possible that *Leishmania* actively promotes M2 polarization of macrophages as a virulence strategy. Of note, a recent report shows that the macrophage response upon visceral infection with *Leishmania infantum* is characterized by an M2b-like phenotype, identified by expression of C-type lectin receptors signature (95).

*Francisella tularensis*, a gram-negative intracellular bacteria and the causative agent of tularemia, induces acute and frequently lethal pulmonary infection. *F. tularensis* replicates in myeloid cells and alveolar epithelial cells in lung of infected mice [reviewed in (96)]. It has been observed that virulent strains induce M2 activation markers on macrophages in wild type but not in IL-4Rα or STAT6 deficient mice (97). Lipid isolated from virulent strain but not from attenuated strain appeared able to induce a TLR2/PPARγ-dependent M2 polarization of macrophage (98), suggesting that redirection of macrophage polarization by *F. tularensis* could constitute an escape immune mechanism.

*Listeria monocytogenes* is a facultative intracellular Gram-positive bacteria able of serious acute infection. *Listeria* infection of macrophages rapidly led to increased expression of PPARγ (99). Selective loss of PPARγ in myeloid cells resulted in enhanced innate immune defense against *Listeria* both, *in vitro* and *in vivo* suggesting that PPARγ-dependent M2 phenotype could favor *L. monocytogenes* multiplication.

*T. gondii* represents one of clear examples of how an intracellular parasite can subvert macrophage polarization to enhance its infectivity. This parasite secretes into the cytoplasm of infected macrophage several virulence factors including a protein, ROP16, displaying protein kinase activity. Remarkably, this protein interferes with the host signaling machinery through the direct phosphorylation of STAT6 (and STAT3), bypassing therefore early cytokine receptor proximal events. Notably, ROP16 was shown to phosphorylate STAT6 on the critical activation residue Tyr641 (39), resulting in the productive transcription of M2-associated genes including Arg1 (41, 100).

In conclusion, numerous evidences suggest that some intracellular bacterial and protozoan pathogens responsible for chronic (*Mycobacteria, Brucella, Leishmania*, and *Toxoplasma*) but also acute (*Francisella* and *Listeria*) infection actively manipulate STAT6-PPARγ/δ pathways to avoid M1 polarization of macrophages and/or benefit from a nutrient rich environment associated to lipid oxidation metabolism. Although the possible impact of macrophage polarization on chronic viral infections remains largely unknown, recent studies suggest that HBV-induced M2 macrophage polarization can participate to immune impairment and pathology (101). Thus, PPARγ/δ pathways hold a pivotal role in the establishment of chronic infection and their ligands may be used as combination therapeutics to limit host pathology or pathogen persistence.

## Beneficial Role of Th2 Response and Alternatively Activated Macrophage during Infection

It is well appreciated that M2 macrophages can act to limit disease severity and protect the host from detrimental effects of an excessive Th1 response, making symbiotic survival between host and parasites more likely. For example, infection of mice or humans with the trematode *S. mansoni* results in a Th2 dependent response. Neutralization of IL-4 or genetic invalidation of IL-4/IL-13 led to increased rates of mortality during acute schistosomiasis, illustrating the protective role of Th2 in this experimental model. Mice rendered selectively deficient for IL-4R in macrophages were extremely susceptible to infection with 100% mortality, suggesting that M2 macrophages are essential during schistosomiasis for protection against organ injury.

In addition, M2 macrophages can also display anti-microbial effector function and actively reduce the level of infection by various pathogens.

Infective larvae of the nematode *N. brasiliensis* enter host animals through skin penetration and further migrate in the lungs and intestine. Primary protective response against *N. brasiliensis* relied mostly on an IL-13-dependent goblet cell hyperplasia leading to increased mucus production in the intestine. Accordingly, IL-4Rα and STAT6 deficient mice failed to expulse the gastrointestinal nematode parasite (102). Interestingly, protective memory response against *N. brasiliensis* implicated neutralization of larva migration from skin to lung (48). Larval trapping was dependent on induction of M2 macrophage differentiation from recruited monocytes as demonstrated by the fact that neutralization of IL-4 or Arg1 abolished larval trapping.

The central pathophysiologic events in severe malaria caused by *Plasmodium falciparum* are the inability of the host defenses to control parasite replication resulting in the excessive release of pro-inflammatory cytokines. Mice treated with an agonist of PPARγ had reduced parasitemia of *Plasmodium* and decreased inflammatory response (103). This effect was mediated by CD36 that served as ligand for *P. falciparum* erythrocyte membrane protein 1 (PfEMP-1) expressed by the parasite (104). CD36-mediated internalization by M2 macrophages facilitated removal of neutrophils and dead tissues to resolve inflammation as well as clearing of the parasite. *P. falciparum* used host hemoglobin as iron source and sequestered heme into a pigment known as hemozoin. It has been showed that hemozoin reacted with membrane phospholipids to generate hydroxy-polyunsaturated fatty acids which are ligands of PPARγ (105), suggesting that the parasite can actively modulate its growth by a PPARγ pathway.

The cestode *Mesocestoides corti* induces a central nervous system infection in mice dominated by M2 macrophages. Absence of STAT6 signaling resulted in enhanced susceptibility to infection coinciding with increased parasite burden in the brain (106) suggesting that M2 macrophages actively control infection.

The ubiquitous fungus *Aspergillus fumigatus* causes invasive and allergenic disease. Host defense relied on the ability of the respiratory immune system to restrict spore germination into invasive hyphae and to limit fungus-induced or inflammation-induced damage in infected tissues. Infection induced a M2 polarization of alveolar macrophages. Control of infection appeared dependent on dectin-1 mediated phagocytosis of fungus by M2 macrophages (107) as dectin-1 deficiency or elimination of M2 macrophages was associated to increased fungal burden in mice.

## Plasticity of Macrophage Polarization during Infection: Two Opposite Examples

Several infectious experimental models demonstrate that immune polarization may display high plasticity, suggesting that M1 and M2 phenotypes may not represent terminally differentiated cells.

Classically, M1 macrophages are implicated in initiating and sustaining inflammation, whereas M2 macrophages differentiate later and are involved in resolution of inflammation and tissue regeneration. In particular, the early and late phases of *Trypanosoma congolense* infection are characterized by M1 and M2 polarization of macrophages, respectively. The shift to M2 appears indispensable to control inflammation and limit tissue injury (105).

However, the opposite scenario has also been reported. *Cryptococcus neoformans*, the etiological agent of cryptococcosis constitutes a well-documented case of M2-to-M1 shift *in vivo*. After inhalation, *C. neoformans* resides primarily in the alveolar spaces, where it can survive and replicate in the extracellular lung environment. The immune response in *C. neoformans*-infected C57BL/6 wild type mice is predominantly Th2 biased and associated with development of M2 macrophages. These latter serve as intracellular reservoirs for the microbe and promote the development of lung pathology (108). In this model, neutralization of IFN-γ resulted in more severe pulmonary infections and lesions (109). In contrast, IL-4 (109) or IL-13 (110) deletion led to Th1-dominated immune responses associated with M1 macrophage granuloma formation displaying iNOS dependent fungicidal activity. Interestingly, the lung immune polarization environment changed overtime (108). Following a strong initial induction of Th2 cytokines in the lungs at 2 and 3 weeks post-infection, a considerable increase in Th1 cytokines occurred at 5 weeks post-infection. This increase in Th1 cytokines was accompanied by a decrease in Th2 cytokines, indicating that the immune system is spontaneously capable of a Th2–Th1 shift. However, if it is now appreciated that polarized T cells exhibit previously unsuspected flexibility and plasticity (111), it remains unclear whether macrophage polarization switches involve recruitment of new precursors or de-differentiation of macrophages *in situ*.

Collectively, these observations suggest that macrophages can undergo dynamic transitions between different functional states. This polarization switch may provide protection to uncontrolled inflammation or constitute an indispensable adaptation to rapid phenotypic change of pathogen during its cycle.

## Conclusions

**Figure 4:**
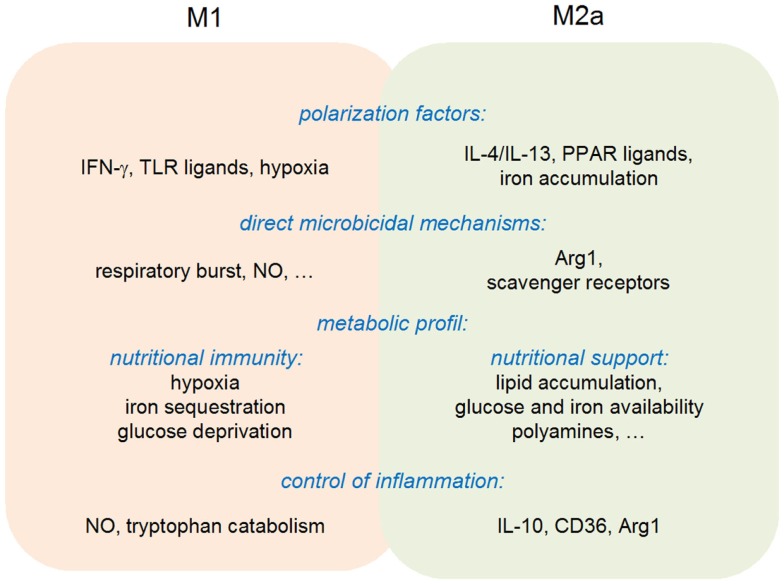
**Multipolar view of M1 and M2a polarization**.

In summary, the available evidences are compatible with the original view of a “division of labor” between M1 and M2 macrophages. M1 macrophages do display an increased microbicidal activity against a wide range of intracellular parasites, while differentiation toward an M2-like state is often observed during the resolution phase of an inflammatory response, favoring in particular tissue repair. This purely dualistic view needs, however, to be considered with care, since M1 macrophages are also able to negatively control an inflammatory response (through notably NO production and IDO1-mediated tryptophan catabolism), while M2 macrophages display anti-microbial activities via expression of Arg1 (Figure 4). The M1/M2 cell paradigm represents, therefore, an additional example of pathogen-tailored immune effectors, endowed with regulatory properties.

A growing number of clinically relevant infectious diseases are characterized by pathogen persistence in the host. Chronic and recurrent infections implicate long-lasting and costly therapy and are the cause of an important morbidity in the world. Therefore, there is a strong interest in understanding the biology of pathogen persistence.

Numerous reports demonstrate that chronic bacterial persistence *in vivo* depends on the ability of bacteria to resist the anti-microbial activity but also to reconfigure local host environment to their profit (112). Pathogens exploit host regulatory pathways limiting damaging inflammatory responses for the benefit of tolerance. They also reprogram host cell metabolism to produce nutrients necessary for their long-term persistence (88, 113). Similar results have been obtained in parasitic infections (12, 89) suggesting that induction of a metabolic shift in infected cells constitutes a general strategy to favor persistence. In this paradigm, PPARs family seems key actor in the hijacking of macrophages by pathogens and an interesting target for therapeutic strategy.

Targeting microbial nutrient requirements represents a promising therapeutic strategy. Both anti-microbial compounds and vaccine have been created to specifically target pathogen iron acquisition systems [reviewed in Ref. (65)]. Unfortunately, microbes often possess redundant mechanisms for nutrient acquisition. A better understanding of these mechanisms may lead to the development of new strategies to control infection.

The importance of nutrient availability in control of infection also leads to focus our attention on the multifactorial and complex impact of modern fat rich diet on chronic infection. Commensal and mutualistic microorganisms are present in all mucosal compartments and form the microbiota. These symbiotic organisms compete with pathogenic microorganisms for nutrients, thereby preventing pathogenic colonization and invasion. As discussed above, obesity can affect macrophage polarization but also drastically change the composition of host microbial community and thus its ability to compete with pathogen for host nutriment availability. A better knowledge of the relationship between nutrition, microbiota, and immune defense could provide guidelines for prophylactic nutritional measures to protect against and/or treat infections.

## Conflict of Interest Statement

The authors declare that the research was conducted in the absence of any commercial or financial relationships that could be construed as a potential conflict of interest.
